# Folic acid alleviates the negative effects of dexamethasone induced stress on production performance in Hyline Brown laying hens

**DOI:** 10.1016/j.aninu.2024.11.011

**Published:** 2024-12-14

**Authors:** Xi Sun, Chaohui Wang, Sijing Li, Xiaoying Liu, Yun Li, Yumeng Wang, Yuxin Niu, Zhouzheng Ren, Xin Yang, Xiaojun Yang, Yanli Liu

**Affiliations:** College of Animal Science and Technology, Northwest A&F University, Yangling 712100, China

**Keywords:** Multi-stressor, Folic acid, Gut microbiota, Laying hen

## Abstract

Multiple stressors are believed to deteriorate production performance and cause substantial economic losses in commercial poultry farming. Folic acid (FA) is an antioxidant compound that can improve oocyte function and regulate gut microbiota composition. The current study was conducted to investigate the role of FA in alleviating stress and improving production performance. Sixty Hyline Brown laying hens at 21 weeks of age were randomly divided into three groups, with 10 replicates in each group and each replicate containing two chickens. Each group received basic diet and saline injection (Con group), basic diet with dexamethasone (DXM) injection (DXM group), or basic diet supplemented with FA (13 mg/kg in the premix) with DXM injection (FA group). The feeding trial lasted five weeks. Birds in the DXM and FA groups receiving subcutaneous DXM injections at a dosage of 4.50 mg/kg per day during the first seven days of the trial. Results showed that the levels of corticosterone, triglyceride, total cholesterol, and malondialdehyde in serum were significantly increased in the DXM group (*P* < 0.05), while the concentrations of FA and 5-methyltetrahydrofolate were decreased in the DXM group (*P* < 0.05). Laying hens in the DXM group had lower laying rates and egg quality, including egg weight, eggshell thickness, eggshell strength, albumen height, and Haugh units (*P* < 0.05). Conversely, FA alleviated these negative impacts. Through transcriptome analysis, a total of 247 and 151 differentially expressed genes were identified among the three groups, and 32 overlapped genes were further identified. Moreover, 44 and 59 differential metabolites were influenced by DXM and FA, respectively. Kyoto Encyclopedia of Genes and Genomes enrichment from the transcriptome and metabolomics showed that the reduced production performance may be due to the disturbance of oocyte production, calcium metabolism, and oxidative stress. Analysis of 16S rRNA gene amplicon sequences revealed the differential microbial composition and potential functional changes among the different groups. LEfSe analysis showed that *Mucispirillum* and *Nautella* were the predominant bacteria in the DXM group, while *Clostridium* was the predominant bacteria in the FA group. Functional prediction demonstrated that stressors enhanced fatty acid biosynthesis, while betaine biosynthesis and retinol metabolism were elevated in the FA group. Dietary FA reversed the elevated levels of bile acids (BA), including cholic acid, taurodeoxycholic acid, and taurochenodeoxycholic acid (*P* < 0.05). The DXM group showed an overall decrease in short-chain fatty acids (SCFA), but FA restored the concentrations of acetic acid, propionic acid, and isobutyric acid (*P* < 0.05). In conclusion, this study reveals that dietary FA can alleviate the degradation of production performance caused by stress through improving circulating antioxidant capacity, maintaining intestinal microbiota homeostasis, and regulating SCFA and BA biosynthesis. Thus, highlighting the prominent role of gut microbe-host interactions in alleviating multi-stresses.

## 1 Introduction

Laying hens are continuously exposed to various stressors in commercial farming, which seriously deteriorate the ultimate production performance of laying hens, and pose a huge economic threat to the poultry industry (Lee et al., 2015, 2022). The breeding process of laying hens involves multiple stressors, including transport stress, cold or heat stress, immune stress, and oxidative stress caused by continuous ovulation. Multi-stressors lead huge changes to metabolism and immunity (Goel et al., 2021); increasing susceptibility to diseases and reducing production performance of laying hens (Jing et al., 2022). Stressors have negative effect on the egg quality, yolk lipids and cholesterol content (Li et al., 2020). Cold or heat stress are believed to cause inflammation (Zhang et al., 2014; Zhao et al., 2013) and damage the liver and intestines of laying hens (Wei et al., 2018; Zhang et al., 2011). Despite several studies elucidating single stress induced suppression on production performance and proposed practicable solutions, many laying hens are still suffering from multi-stressors. Consequently, there are substantial unmet needs for laying hens that are subjected to multiple stressors.

Glucocorticoids are a type of steroid hormones that respond rapidly to stressor stimuli (Alba et al., 2019; Häffelin et al., 2020). Dexamethasone (DXM), a synthetic glucocorticoid, is similar to the glucocorticoids secreted by animals under multiple stress conditions (Gao et al., 2008; Lin et al., 2004), and has been widely used as a glucocorticoid analogue (Chang et al., 2015). In addition, DXM has been extensively used to establish stress models for chickens (Osho and Adeola, 2020; Pan et al., 2019; Su et al., 2021; Zhai et al., 2020; Zhou et al., 2019). Thus, DXM can serve as an effective stress-inducing agent for exploring the underlying mechanisms behind the decline in egg production in laying hens resulting from exposure to multiple stressors.

Recent studies have demonstrated that folic acid (FA) functions as an antioxidant compound, reducing levels of reactive oxygen species and endoplasmic reticulum stress, and preventing oxidative stress-induced cell apoptosis (Cui et al., 2018;Zhang et al., 2023b). Folic acid has been shown to improve oocyte function and fetal intrauterine development (Fu et al., 2021; Jing et al., 2014). Additionally, dietary supplementation with FA (1.5 mg/kg) enhances the antioxidant status of broilers under heat stress (Gouda et al., 2020). Certain microorganisms in the cecum, such as *Fusobacteria* and *Proteobacteria* (Magnúsdóttir et al., 2015), can synthesize FA, which play an important role in intestinal homeostasis and host energy metabolism (Bai et al., 2021). Therefore, we hypothesized that dietary FA may increase circulating antioxidant capacity, maintain gut microbiota homeostasis and promote the generation of short-chain fatty acids (SCFA) to alleviate the decline in production performance caused by multiple stress. The current study was carried out to explore the adverse effects of multiple stressors on production performance and the potential effects of FA as a promising nutritional additive to alleviate stress in laying hens. We systematically elucidated this through DXM-modeled multiple stressors and multi-omics analyses. Our results provide evidence that dietary FA supplementation improves production performance in laying hens under multiple stress conditions, and highlight the prominent role of interactions between microbes and hosts in alleviating stress.

## 2 Materials and methods

### 2.1 Animal ethics statement

The animal management and experimental procedures in this study were performed in accordance with the Guidelines for Care and Use of Laboratory Animals and have been approved by the Animal Ethics and Welfare Committee of Northwest A&F University (protocol number DK2022007).

### 2.2 Animal treatments, sampling

Sixty Hyline Brown layers (21-week-old) were allocated into three groups: (1) a control group receiving basic diet with saline injection (Con group), (2) a group receiving basic diet with DXM injection (DXM group), (3) a group receiving basic diet supplemented with FA in addition to DXM injection (FA group), with 10 replicates in each group, each replicate containing two chickens. The premix of the FA group contains 13 mg/kg of FA, which is 10 times the FA content in the premix of basic diet; the dosage based on our previous studies (Liu et al., 2022, 2024a). Detailed composition of the basic is outlined in Table 1. After adapting to the environment, the laying hens in the DXM and FA groups were subcutaneously injected with DXM at a dose of 4.5 mg/kg per day for seven days (Liu et al., 2024a; Sun et al., 2024a, Sun et al., 2024b). The experiment lasted for five weeks: following the administration of DXM for one week, the birds were fed their respective diets for the next four weeks. Before euthanasia of laying hens, blood samples were collected from the brachial vein. A small piece of liver and cecal contents were collected, rapidly frozen using liquid nitrogen, and subsequently stored at –80 °C for further analysis.

**Table 1:** Composition and nutrient levels of basic diet for Hyline Brown laying hens (%).

Item	Content
**Composition (air-dry basis)**	
Corn	56.69
Distillers dried grains with solubles	4.00
Soybean meal, 43%	25.77
DL-Methionine, 98.5%	0.18
Soybean oil	1.51
Calcium carbonate	9.04
Dicalcium phosphate	1.15
Sodium chloride	0.26
Choline chloride, 60%	0.15
Bentonite	0.25
Premix^1^	1.00
Total	100.00
**Calculated nutrient levels**	
Metabolizable energy, kcal/kg	2600
Crude protein	16.50
Total phosphorus	0.49
Calcium	3.50
**Analyzed nutrient levels**	
Gross energy, kcal/kg	2951
Crude protein	16.61
Crude fiber	29.95
Total phosphorus	0.53
Calcium	3.52

1 Provided per kilogram of diet: iron, 60 mg; manganese, 60 mg; copper, 8 mg; zinc, 80 mg; selenium, 0.3 mg; iodine, 0.35 mg; vitamin A, 8000 IU; vitamin D_3_, 1600 IU; vitamin E, 30 mg; menadione, 1.5 mg; vitamin C, 200 mg, thiamine, 4 mg; riboflavin, 13 mg; pantothenic acid, 15 mg; nicotinamide, 20 mg; pyridoxine, 6 mg; biotin, 0.15 mg; folic acid (FA), 1.3 mg; cobalamin, 0.02 mg.

### 2.3 Chemical composition of the diet

All birds were housed in a climate-controlled henhouse and were given unrestricted access to their respective diets and water at the Experimental Teaching Center of Animal Science at Northwest A&F University. The ambient temperature was maintained at 22 to 24 °C, and the lighting regimen followed a 12-h cycle. The diets were formulated according to the recommended level of Chinese Feeding Standard of Chicken (NY/T 33-2004, Ministry of Agriculture of the People’s Republic of China, 2004) and NRC (1994). The dietary metabolizable energy (ME) was calculated according to the following formula: ME = corn × ME1 + DDGS × ME2 + soybean meal × ME3 + … … + premix × ME11. All values from ME1 to ME11 were based on the 32nd edition Chinese Feed Composition and Nutritional Value Table (Xiong, 2021). The other calculated nutritional values in Table 1 are referred to the 32nd edition Chinese Feed Composition and Nutritional Value Table (Xiong, 2021). The crude fiber content in the diet was measured according to accepted methodologies outlined by the Association of Official Agricultural Chemists, using the Weende method 978.10 (AOAC, 2006). The gross energy was analyzed using an oxygen bomb calorimeter (1341 Calorimeter, Parr Instrument Company, USA), following the steps described by the method 9831 (ISO, 1998). The crude protein (N × 6.25) content was determined using the Kjeldahl method 990.03 (AOAC, 1995) with a nitrogen analyzer (Leco CNS-2000 analyzer, MO, USA). With reference to the previous methodology, the actual phosphorus content in the diet was analyzed using a spectrophotometer (UV-2700, Shimadzu, Japan) in accordance with China National Standard (GB/T 6437-2018), while the calcium content was measured using flame atomic absorption spectrophotometry (Zeenit700P, Analytik Jena, Germany) according to China National Standard (GB/T 6436-2018). The samples were ashed at 600 °C for 12 h using a muffle furnace, and the minerals were determined using inductively coupled plasma mass spectrometry (Varian ICP-OES Vista Pro, Spectralab Scientific Inc., Canada).

### 2.4 Production performance and egg quality characteristics

To assess production performance over four consecutive weeks, eggs were collected from each week and analyzed for egg quality parameters. In the last two weeks, the number of eggs laid in each group was counted daily to calculate the egg production rate. In brief, we utilized a texture analyzer (EFG-0503, Robotmation, Japan) to assess eggshell strength, a dial pipe gauge (ETG-1061, Robotmation, Japan) to gauge eggshell thickness after removing the inner membranes, and a multifunctional egg quality analyzer (EMT-5200, Robotmation, Japan) to evaluate the egg internal quality, including albumen height and Haugh unit.

### 2.5 Serum biochemical and antioxidant measurements in the liver

The blood samples were centrifuged at 3000 × *g* for 10 min to obtain serum. The relevant indexes of serum lipid metabolism, including triglyceride, total cholesterol, high density lipoprotein cholesterol (HDL-C), low density lipoprotein cholesterol (LDL-C), and biomarkers of liver injury, including total bilirubin, total bile acids, aspartate aminotransferase (AST), lactate dehydrogenase (LDH), were detected using the Hitachi-7180 automatic biochemical analyzer at Yangling Demonstration Zone Hospital. Cold saline was added to liver tissue and homogenized on ice, and then centrifuged at 8000 × *g* for 15 min. Total protein in the tissue supernatant and serum were unified using the BCA kit (AccuRef Scientific Co., Ltd., Xi'an, China). Moreover, the concentrations of malondialdehyde (MDA) and total anti-oxidation capacity in both liver and serum were determined using commercial kits (Nanjing Jiancheng Bioengineering Institute, Nanjing, China), respectively. Furthermore, the abundance of FA and 5-methyltetrahydrofolate (5-MTHF) in the liver, serum and serum corticosterone (CORT) were detected using commercial ELISA kits based on the instructions of the kits (Jiangsu Meibiao Biotechnology Co., Ltd, China).

### 2.6 Transcriptome profiling

Total RNA was extracted from the collected liver samples by the RNAex Pro kit (AG21102, AG, China). RNA concentration and purity were evaluated using the BioAnalyzer 2100 (Nanodrop ND-1000, Thermo Fisher Scientific). RNA sequencing libraries for each group were prepared (Shanghai Personal Biotechnology Co., Ltd., China) and further sequenced on the Illumina NextSeq 500 (Illumina, Inc., USA). The raw data was initially filtered, and quality control performed to eliminate low-quality reads through SOAPnuke. Afterwards, the clean reads were aligned to the chicken genome assembly (GRCg7b, https://www.ncbi.nlm.nih.gov/genome/111) via HISAT2. Gene expression was quantified with RSEM software by calculating fragments per kilobase per million. Principal component analysis and differentially expressed genes (DEG) were identified using the DESeq2 (v.1.18.1) software based on a threshold set to log2 fold change >1 and *P* < 0.05. The heatmap R packages were applied to map clustering among different groups. Subsequently, the identified DEG were annotated to the KEGG (http://www.kegg.jp/), and KEGG enrichment analyses and visualization were conducted through Clusterprofiler based on *P* < 0.05. Detailed process steps for RNA-sequence were based on a previous study (Cogburn et al., 2018).

### 2.7 Serum untargeted metabolomics

The metabolomics analysis was conducted (Personal Biotechnology Co., Ltd., China) Briefly, 300 μL of methanol and 10 μL of internal standard were added to a centrifuge tube containing 100 μL of collected serum to precipitate protein. The 200 μL supernatant obtained by centrifugation was transferred to a sample bottle for subsequent measurement. The derivative samples were further analyzed using liquid chromatography-mass spectrometry (LC-MS) by coupling the ultrahigh performance liquid chromatography (UHPLC) System (Thermo Fisher Scientific, USA) with the Thermo Scientific Q-Exactive high-accuracy mass spectrometer. The chromatographic and mass spectrometry conditions were as previously described (Deng et al., 2022). Subsequently, based on a previous report (Li et al., 2017), the raw data was generated through Compound Discoverer 3.1 to identify and select peaks, and perform library comparison. Based on the redundant m/z peaks, the molecular mass was defined. Orthogonal partial least-squares discriminant analysis (OPLS-DA) was performed to eliminate the effect of variability using Ropls software. Then, differential metabolites were selected based on a threshold of variable importance in the projection (VIP) >1.0, *P* < 0.05, and |FC| > 2. The pathway enrichment of differential metabolites was constructed using MetaboAnalyst software, and further annotated to the KEGG database. Differential metabolic pathways were displayed using the KEGG pathway mapper function and represented using bubble charts.

### 2.8 Microbiota DNA extraction and 16S rRNA analysis

Genomic DNA was extracted from cecal microbiota using a commercial DNA kit (Tiangen, Beijing, China). The 16S rRNA gene was amplified targeting the hypervariable regions V3–V4 of bacteria with the following primers: F: 5'-ACTCCTACGGGAGGCAGCA-3', R: 5'-GGACTACHVGGGTWTCTAAT-3', as previously described (Liu et al., 2023). The resulting product was purified with the Agencourt AMPure Beads (Beckman Coulter, Indianapolis, IN, USA), and subsequently subjected to library construction. Then, 16S rRNA sequencing was performed on the Illumina platform (San Diego, USA) by Shanghai Personal Biotechnology Co., Ltd. (China).

The raw data after quality filtering was compared to the Greengene 13 database for further analysis. Sequence length distribution was counted, and taxonomic components were visualized using QIIME2 (http://qiime.org/index.html). Principal component analysis based on Bray–Curtis was used to estimate the heterogeneity of community structure. Alpha and beta diversity were analyzed through QIIME2 and principal coordinate analysis. Additionally, LEfSe analysis was conducted with the following threshold: linear discriminant analysis (LDA) > 2 and *P* < 0.05, to detect differentially abundant microorganisms. Phylogenetic investigation of communities by reconstruction of unobserved states was applied to predict microbial functional changes, which were further annotated to the KEGG database. The functional differences were visualized using STAMP software (Zhang et al., 2022).

### 2.9 Serum bile acids (BA) and cecal SCFA assessment

The composition and concentration of serum BA were detected using LC-MS spectrometry. To precipitate the protein, serum samples were added with 320 μL of acetonitrile containing the internal standards were centrifuged at 8000 × *g* at 4 °C for 10 min. The supernatant was further transferred to a sampling vial. Dried samples were redissolved in 100 μL of methanol/water (30:70, v/v). The LC-MS analysis was performed using a HESI source (Thermo Fisher Scientific, San Jose, CA, USA) on Ultimate 3000 UHPLC and Q-Exactive mass spectrometer. Chromatographic separation was performed on UPLCTM BEH C18 column (Waters, USA). The detailed experimental process described in the previous report (Yan et al., 2023).

Gas chromatography-mass spectrometry (GC–MS) was used to detect the concentrations of SCFA cecal chyme. Initially, 0.3 g of cecal contents were homogenized in cold normal saline, followed by centrifugation at 10,000 × *g* for 10 min at 4 °C. The supernatant was obtained and mixed with metaphosphoric acid, and the sample residue was further extracted with 200 μL of methanol/acetonitrile (2:1, v/v) containing internal standards. After 4 h of quiescence at 4 °C, the mixture was centrifuged at 10,000×*g* at 4 °C for 15 min, and crotonic acid was added to the supernatant. The pre-treated samples were then transferred to meteorological vials for GC–MS analysis, with parameters set according to reported methods (Song et al., 2020). The peaks of acetic acid, propionic acid, isobutyric acid, butyric acid, and valeric acid were measured, and their concentrations were calculated according to the standard curve.

### 2.10 Statistical analyses

In the GLM program of SPSS 27 (SPSS Inc., Chicago, IL), one-way ANOVA, normal distribution test, and homogeneity of variance (Levene test) were performed to analysis the data. The statistical analytical model used in this study was as follows:

*Y*_*ij*_ = *μ* + *T*_*i*_ + *S*_*j*_ + *ε*_*ij*_,

where *i* represents group *i*, *j* represents the *j* th observation; *μ* was the overall mean; *T*_*i*_ was the fixed effect of treatment; *S*_*j*_ was the random effect; *ε*_*ij*_ is the observation residual error, and *Y*_*ij*_ was the observation of dependent variables. The Duncan's multiple comparisons was used to determine significant differences among different treatments if the variance meets the homogeneity test. The *P*-value <0.05 was considered to have statistical significance (∗*P* < 0.05, ∗∗*P* < 0.01). The bar chart was created through GraphPad Prism 8 (Boston, USA).

## 3 Results

### 3.1 Phenotype identification for production performance of laying hens

To interrogate the impact of multiple stressors on production performance and the potential role of dietary FA supplementation, a comparison of egg production rate and egg quality indicators was conducted (Table 2). There was no significant difference in body weight between groups at the beginning and end of the experiment. However, the administration of DXM significantly reduced the laying rate and negatively impacted egg weight, eggshell thickness, eggshell strength, albumen height, and Haugh unit (*P* < 0.001 or *P* < 0.05). Conversely, FA supplementation alleviated these negative impacts (*P* < 0.05). These results demonstrate that the multiple stressors simulated by DXM lead to a decrease in laying rate and egg quality of laying hens, which confirms the success of the stress model construction.

**Table 2:** Growth performance and egg production performance of Hyline Brown laying hens 1.

Item	Treatment groups^2^			SEM	*P*-value
	Con	DXM	FA		
Initial body weight, kg	1.71	1.62	1.68	0.029	0.474
Final body weight, kg	1.94	1.88	1.92	0.030	0.686
Average feed intake, g	105.28	96.45	94.18	2.271	0.090
Laying rate, %	96.67^a^	74.07^c^	84.26^b^	2.388	<0.001
Egg weight, g	59.84^a^	53.78^c^	57.80^b^	0.356	<0.001
Eggshell strength, kg/cm^2^	6.01^a^	5.48^b^	5.75^b^	0.075	0.017
Eggshell thickness, mm	0.43^a^	0.41^b^	0.42^ab^	0.002	0.001
Albumen height, mm	9.35^a^	8.69^b^	9.32^a^	0.077	<0.001
Haugh units	96.19^a^	94.41^b^	95.85^a^	0.315	0.005

DXM = dexamethasone; FA = folic acid.

1 The different superscript letters a, b, and c represent significant differences between different groups (*P* < 0.05) (egg quality data *n* = 60, other data *n* = 10).

2 Treatment groups: (1) a control group receiving basic diet with saline injection (Con group), (2) a group receiving basic diet with DXM injection (DXM group), (3) a group receiving basic diet supplemented with FA in addition to DXM injection (FA group).

### 3.2 Serum biochemical and antioxidant indicators

As depicted in Table 3, the CORT was significantly elevated in the DXM group, while it has reverse trend in the FA group (*P* = 0.054). Serum indicators reflecting lipid metabolism, such as total cholesterol and triglyceride were upregulated under stress conditions (*P* = 0.048 and *P* = 0.006), but had no significant effect on HDL-C. Folic acid significantly reduced serum total cholesterol levels (*P* = 0.048), but had no effect on other lipid metabolism related biochemical indicators. Moreover, FA reversed the increase levels of the total bilirubin and total bile acids (*P* = 0.003 and *P* = 0.039), which are associated with liver injury. There was no significant difference in serum AST and LDH. Folic acid alleviated the increase in serum MDA levels caused by DXM (*P* < 0.05), but there were no significant changes in other serum and liver antioxidant indicators. Furthermore, both liver and serum decreased FA concentration and were up-regulated in the FA group (*P* = 0.030 and *P* = 0.045), and 5-MTHF in serum showed the same trend (*P* = 0.032). There was no significant change in hepatic 5-MTHF.

**Table 3:** Effects of dietary FA on biochemistry indicators, antioxidant indicators, and FA content of serum and liver in Hyline Brown laying hens^1^.

Item	Treatment groups^2^			SEM	*P*-value
	Con	DXM	FA		
**Serum**					
CORT, ng/mL	156.31^b^	174.09^a^	160.15^ab^	4.196	0.054
Total cholesterol, mmol/L	3.58^b^	4.45^a^	3.72^b^	0.168	0.048
Triglyceride, mmol/L	15.17^b^	21.50^a^	18.50^ab^	0.886	0.006
HDL-C, mmol/L	0.35	0.34	0.34	0.021	0.956
LDL-C, mmol/L	0.31	0.40	0.35	0.018	0.172
Total bilirubin, μmol/L	1.90^b^	3.96^a^	2.65^b^	0.288	0.003
Total bile acids, μmol/L	25.57^b^	35.11^a^	22.64^b^	2.105	0.039
AST, U/L	225.22	208.33	208.33	5.275	0.333
LDH, U/L	761.44	881.33	828.30	52.335	0.672
T-AOC, mg/mL	1.48	1.46	1.66	0.124	0.798
MDA, mg/mL	16.03^b^	31.01^a^	11.58^b^	2.739	0.008
FA, μmol/L	25.32^a^	25.14^b^	25.30^a^	0.034	0.045
5-MTHF, μg/L	214.01^a^	212.50^b^	213.69^a^	0.258	0.032
**Liver**					
T-AOC, mmol/g prot	0.43	0.47	0.51	0.041	0.754
MDA, mmol/mg prot	1.53	1.63	1.43	0.058	0.360
FA, μg/g prot	1.94^ab^	1.92^b^	1.95^a^	0.005	0.030
5-MTHF, ng/g prot	13.19	13.18	13.22	0.020	0.673

FA = folic acid; DXM = dexamethasone; CORT = corticosterone; HDL-C = high density lipoprotein cholesterol; LDL-C = low density lipoprotein cholesterol; AST = aspartate aminotransferase; LDH = lactate dehydrogenase; T-AOC = total anti-oxidation capacity; MDA = malondialdehyde; 5-MTHF = 5-methyltetrahydrofolate.

1 The different superscript letters a and b represent significant differences between different groups (*n* = 10, *P* < 0.05).

2 Treatment groups: (1) a control group receiving basic diet with saline injection (Con group), (2) a group receiving basic diet with DXM injection (DXM group), (3) a group receiving basic diet supplemented with FA in addition to DXM injection (FA group).

### 3.3 Transcriptome identifies gene expression pattern and pathway changes

**Fig. 1:**
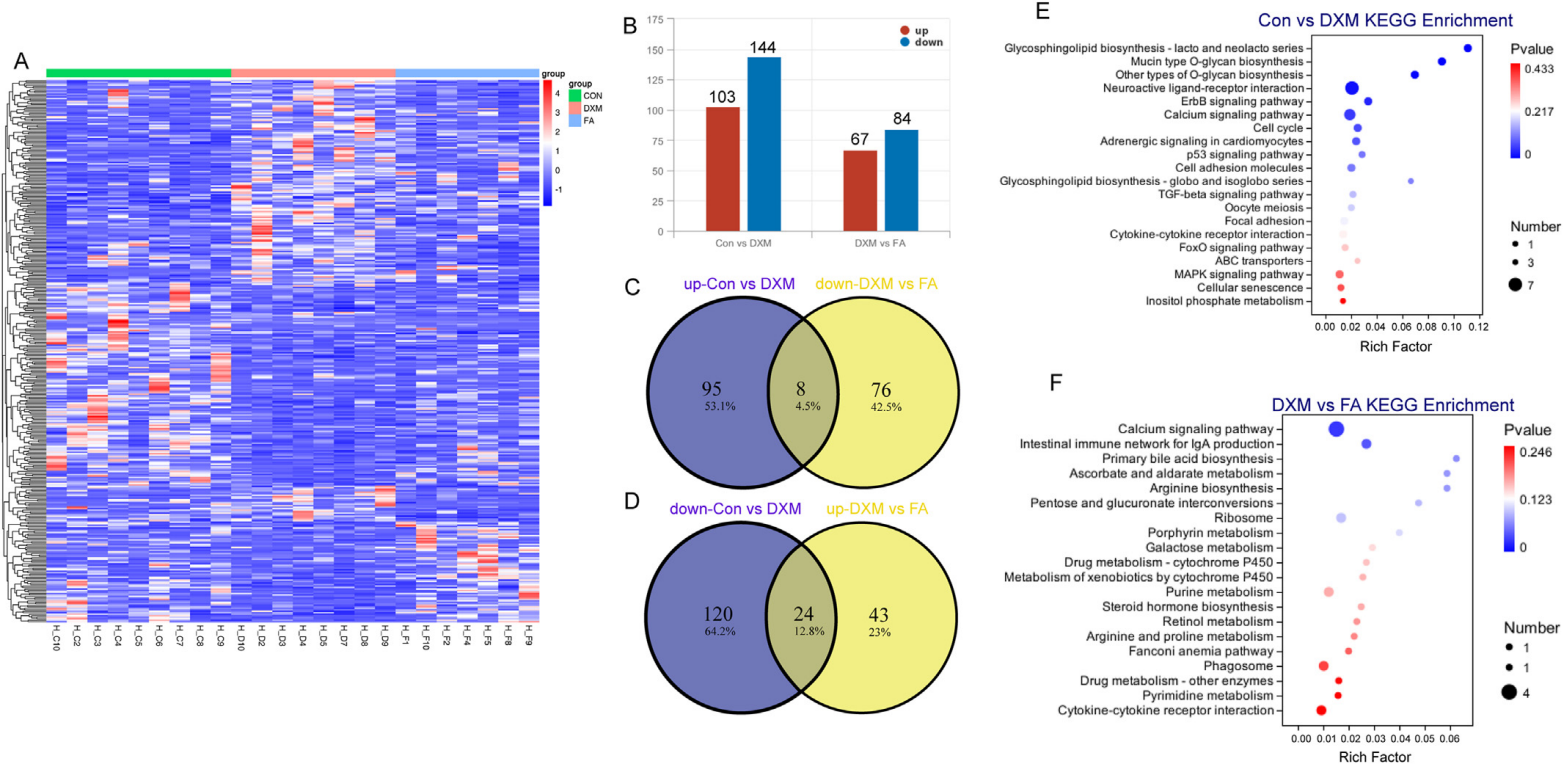
Identification of DEG and KEGG enrichment from transcriptomics in Hyline Brown laying hens. (A) DEG heat map of the liver. Higher expression genes are shown in red color, while lower genes are presented in blue color. (B) The identified up- or down-regulated DEG in different groups. (C-D) Venn diagram for overlapping DEG between Con vs. DXM and DXM vs. FA groups. (E-F) KEGG pathway enrichment from transcriptomics. Total DEG were used for the enrichment analysis for Con vs. DXM groups and DXM vs. FA groups. Treatment groups: (1) a control group receiving basic diet with saline injection (Con group), (2) a group receiving basic diet with DXM injection (DXM group), (3) a group receiving basic diet supplemented with FA in addition to DXM injection (FA group). DEG = differentially expressed genes; KEGG = Kyoto Encyclopedia of Genes and Genomes; DXM = dexamethasone; FA = folic acid.

To further evaluate the changes in transcriptional levels after exposure to multi-stressors, transcriptomics was carried out to screen for DEG and pathways (Fig. 1). The heatmap of DEG cluster assays revealed that some genes exhibited gradual increases or decreases after exposure to multiple stressors, while others exhibited reversal effects by adding FA (Fig. 1A). The number of up- and down-regulated DEG between the Con and DXM groups were 103 and 144, respectively. Additionally, 67 up- and 84 down-regulated DEG were identified between the DXM and FA groups (Fig. 1B). Among the upregulated DEG between the Con and DXM groups, eight genes that were downregulated by FA addition through the Venn diagram. Simultaneously, 24 DEG were downregulated after experiencing multi-stressors, but were rescued in the FA group (Fig. 1C and D). The KEGG pathway analysis revealed that the DEG from Con vs. DXM groups were enriched in the calcium signaling pathway, transforming growth factor-β signaling pathway, oocyte meiosis, inositol phosphate metabolism, cellular senescence and MAPK signaling pathways. Moreover, the calcium signaling pathway, steroid hormone biosynthesis and primary BA biosynthesis were enriched in the DXM vs. FA groups (Fig. 1E and F).

### 3.4 Identification of metabolic profiles and potential biomarkers related to oxidative stress during stress processes

**Fig. 2:**
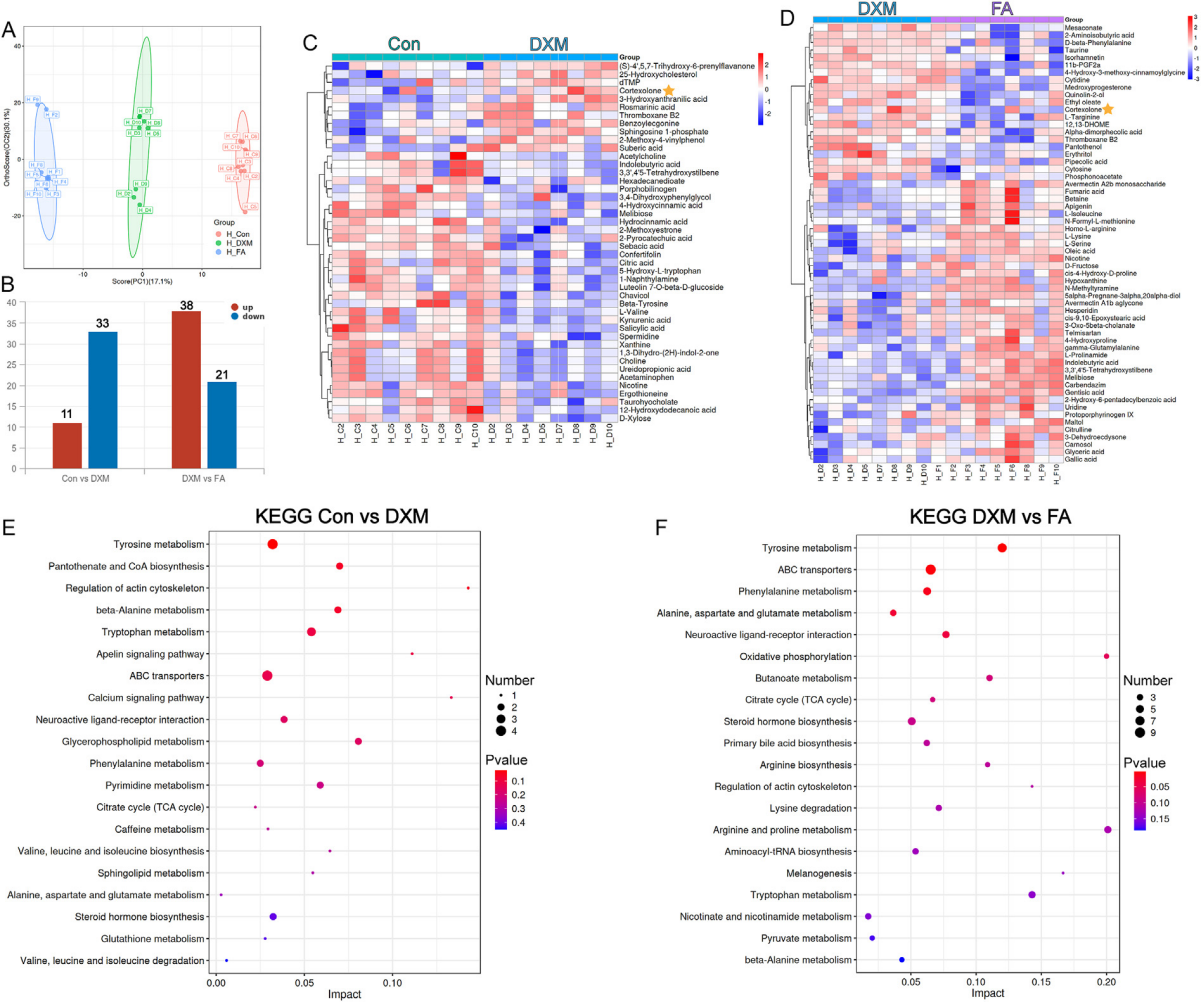
Differential metabolites and KEGG analyses of serum metabolomics in Hyline Brown laying hens. (A) Orthogonal partial least-squares discriminant analysis score plots based on identified metabolites. (B) The number of upregulated or downregulated differential metabolites in different groups. (C and D) Heat maps of differential metabolites, presented as Con group vs. DXM group and DXM group vs. FA group, respectively. Metabolites with higher abundance were shown in red, whereas lower metabolites were presented in blue. (E-F) KEGG functional enrichment analysis. The pathways enriched from all differential metabolites between Con group vs. DXM group and DXM group vs. FA group. The color and size of each circle are based on *P*-values and pathway impact values, respectively. Treatment groups: (1) a control group receiving basic diet with saline injection (Con group), (2) a group receiving basic diet with DXM injection (DXM group), (3) a group receiving basic diet supplemented with FA in addition to DXM injection (FA group). OPLA-DA = orthogonal partial least-squares discriminant analysis; KEGG = Kyoto Encyclopedia of Genes and Genomes; DXM = dexamethasone; FA = folic acid.

The occurrence of oxidative stress is always following experiencing stressors. Results indicated significant separation between the Con, DXM, and FA groups (Fig. 2A), with a total of 44 and 59 differential metabolites were identified in Con vs. DXM and DXM vs. FA groups (Fig. 2B). The heatmap of metabolite abundance also demonstrated clear separation of each group (Fig. 2C and D). Notably, the DXM group exhibited a higher level of cortexolone, which was alleviated in the FA group (Table S1 and S2). Moreover, the abundance of betaine was significantly increased in the FA group compared to the DXM group, which was consistent with the pathway enrichment from the transcriptome analysis. Detailed information regarding the metabolites is listed in supplemental Table S1 and S2. Pathway enrichment showed that tyrosine metabolism exhibited the most significant changes in both Con vs. DXM groups and DXM vs. FA groups. Furthermore, the differential metabolites between the Con and DXM groups were enriched in pathways related to production performance (steroid hormone biosynthesis and calcium signaling pathways), as well as stress-related pathways (tyrosine metabolism, beta-Alanine metabolism, apelin signaling pathway, caffeine metabolism, and glutathione metabolism) (Fig. 2E). The differential metabolite enriched pathways between the DXM and FA groups were related to production performance and oxidative stress, including oxidative phosphorylation, steroid hormone biosynthesis, nicotine and nicotinamide metabolism, beta-alanine metabolism, as well as butyric acid metabolism and primary BA biosynthesis, which were closely related to intestinal homeostasis (Fig. 2F).

### 3.5 Differential microbiological and functional analyses of the cecum in laying hens

**Fig. 3:**
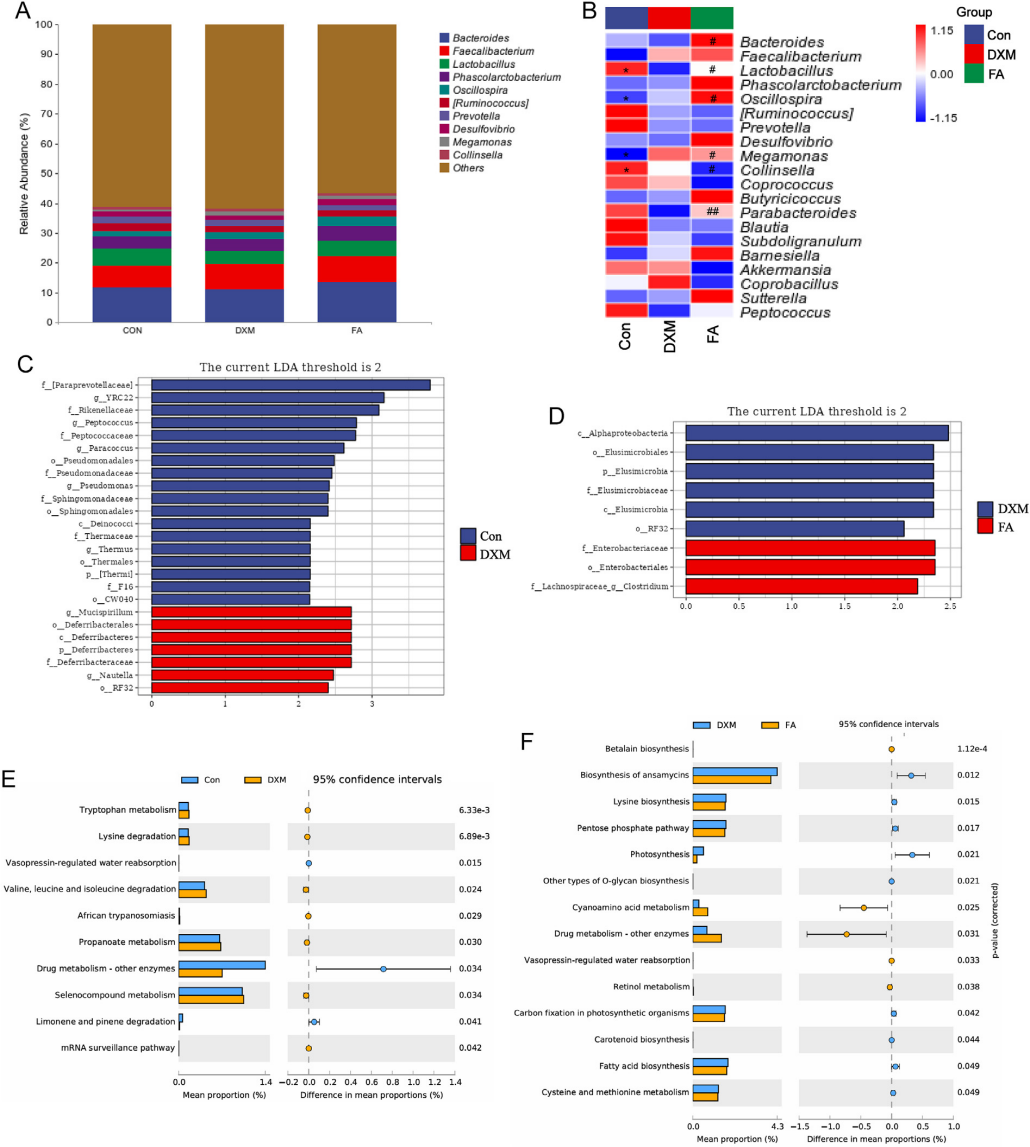
Differential microbiological and functional analysis of the cecum in Hyline Brown laying hens. (A) Relative abundance of bacterial composition at the genus level of cecal microbiota. (B) Heat map of differential expression of microorganisms in the top 20 for expression abundance at the genus level. ∗*P* < 0.05 and ∗∗*P* < 0.01 denote the statistical significance between Con vs DXM groups, while ^#^*P* < 0.05 and ^##^*P* < 0.01 denote the statistical significance between DXM vs FA groups. (C and D) LEfSe analyzed the differences in microbial abundance between Con group vs. DXM group, as well as DXM group vs. FA group. The default parameters used were LDA score >2 and *P* < 0.05. (E and F) Functional enrichment analysis of secondary metabolic pathways related to changes in cecal microbiota. Treatment groups: (1) a control group receiving basic diet with saline injection (Con group), (2) a group receiving basic diet with DXM injection (DXM group), (3) a group receiving basic diet supplemented with FA in addition to DXM injection (FA group). LEfSe = linear discriminant analysis effect size; DXM = dexamethasone; FA = folic acid.

As shown in Fig. 3A, the abundance of *Bacteroides*, *Faecalibacterium*, and *Lactobacillus* were ranked as the top 3 abundant genera. The heatmap of the top 20 microorganisms at the genus level revealed that FA significantly restored the decreased *Lactobacillus* level in the DXM group (Fig. 3B). The dominant bacteria at the genus level of the Con group were *YRC22*, *Peptococcus*, *Paracoccus*, *Pseudomonas*, and *Thermales*, while *Mucispirillum* and *Nautella* were found to be predominant in the DXM group. *Clostridium* was higher in the FA group (Fig. 3C and D). Functional prediction of cecal microbiota at level 3 showed enhanced lysine, valine, leucine, and isoleucine degradation, enhanced metabolism of tryptophan, propanoate metabolism, drug metabolism-other enzymes, and selenocompound metabolism in the DXM group (Fig. 3E). Furthermore, between DXM vs. FA groups, biosynthesis of ansamycins, lysine biosynthesis, pentose phosphate pathway, photosynthesis, other types of O-glycan biosynthesis, carbon fixation in photosynthetic organisms, carotenoid biosynthesis, FA biosynthesis, cysteine and methionine metabolism were enriched in the DXM group, while functional capacities involved in betalain biosynthesis, cyanoamino acid metabolism, drug metabolism-other enzymes and retinol metabolism were found in the FA group (Fig. 3F).

### 3.6 Serum BA and cecal SCFA changes following stress

To investigate the potential interaction between the gut microbiota and the host, the current study further analyzed the levels of serum BA and cecal SCFA (Table 4, Table 5). The analysis of serum BA revealed a significant increase in primary BA (*P* < 0.05), including chenodeoxycholic acid (CDCA), cholic acid (CA), taurocholic acid (TCA), and taurochenodeoxycholic acid (TCDCA), after DXM injection (Table 4). However, dietary FA mitigated the increase in CA and TCDCA (*P* < 0.001 and *P* = 0.011). Additionally, the levels of serum secondary BA, taurodeoxycholic acid (TDCA), was significantly elevated in the DXM group, with TDCA being notably reduced in the FA group (*P* = 0.001). As illustrated in Table 5, the DXM group exhibited a general decline in SCFA levels, encompassing acetic acid, propionic acid, butyric acid, isobutyric acid, valeric acid, and isovaleric acid (*P* < 0.05). Moreover, the concentration of acetic acid, propionic acid, and isobutyric acid in the cecum was significantly restored in the FA group (*P* < 0.05).

**Table 4:** Effects of dietary folic acid on serum bile acids in Hyline Brown laying hens (nmol/L)^1^.

Item	Treatment groups^2^			SEM	*P*-value
	Con	DXM	FA		
CDCA	28.89^b^	81.76^a^	43.36^ab^	8.796	0.033
GCDCA	0.68	0.68	0.68	0.002	0.274
CA	0.16^b^	0.20^a^	0.17^b^	0.005	<0.001
TCA	1.13^b^	4.30^a^	2.81^a^	0.414	0.001
GCA	0.04^a^	0.04^b^	0.04^b^	0.001	<0.001
TCDCA	1986.25^b^	2744.03^a^	2222.00^ab^	124.769	0.011
TDCA	0.67^b^	0.92^a^	0.71^b^	0.034	0.001
UDCA	0.16	0.16	0.16	0.002	0.130
TLCA	0.54	0.57	0.55	0.005	0.180
DCA	1.01	0.81	0.88	0.047	0.106

DXM = dexamethasone; FA = folic acid; CDCA = chenodeoxycholic acid; GCDCA = glycochenodeoxycholic acid; CA = cholic acid; TCA = taurine-conjugated cholic acid; GCA = glycocholic acid; TCDCA = taurochenodeoxycholic acid; TDCA = taurodeoxycholic acid; UDCA = ursodeoxycholic acid; TLCA = taurolithocholic acid; DCA = deoxycholic acid.

1 The different superscript letters a and b represent significant differences between different groups (*n* = 10, *P* < 0.05).

2 Treatment groups: (1) a control group receiving basic diet with saline injection (Con group), (2) a group receiving basic diet with DXM injection (DXM group), (3) a group receiving basic diet supplemented with FA in addition to DXM injection (FA group).

**Table 5:** Effects of dietary FA on cecal SCFA in Hyline Brown laying hens (μmol/g)^1^.

Item	Treatment groups^2^			SEM	*P*-value
	Con	DXM	FA		
Acetic acid	20.60^a^	14.90^b^	19.95^a^	0.964	0.022
Propionic acid	6.07^a^	3.85 ^b^	5.82^a^	0.319	0.003
Butyric acid	12.46^a^	6.38 ^b^	7.76 ^b^	0.775	0.002
Isobutyric acid	5.55^a^	4.38 ^b^	4.62^a^	0.166	0.007
Valeric acid	6.14^a^	4.19 ^b^	4.73 ^b^	0.283	0.011
Isovaleric acid	1.01	0.81	0.88	0.047	0.205

SCFA = short-chain fatty acids; DXM = dexamethasone; FA = folic acid.

1 The different superscript letters a and b represent significant differences between different groups (*n* = 10, *P* < 0.05).

2 Treatment groups: (1) a control group receiving basic diet with saline injection (Con group), (2) a group receiving basic diet with DXM injection (DXM group), (3) a group receiving basic diet supplemented with FA in addition to DXM injection (FA group).

## 4 Discussion

Multiple stressors often occur during the breeding process of laying hens, which significantly affect production performance and mortality rates in poultry (Goel et al., 2021; Jing et al., 2022). However, the precise pathogenesis of laying hens after exposure to multi-stressors remains largely unknown. Therefore, revealing the mechanism of multiple stressors may provide potential targets and intervention methods for laying hens to cope with the changing environments. Recent studies have demonstrated that FA can balance oxidative stress (Cui et al., 2018), maintain intestinal homeostasis (Liu et al., 2022) and enhance egg quality (Bagheri et al., 2019; Jing et al., 2014). However, whether FA exhibits a beneficial effect in laying hens exposed to multiple stressors remains ambiguous. In this study, DXM was used to simulate a multiple stressors model, and employed multi-omics to elucidate the internal factors contributing to decreased egg production performance, and the potential beneficial effects of dietary nutrient FA. The results suggest a novel approach for ameliorating the reduction in production performance of laying hens in intensive conditions.

Glucocorticoids are a class of steroid hormones that respond quickly to stressor stimuli. However, elevated levels of circulating glucocorticoids can inhibit the production of luteinizing hormones and progesterone, resulting in a decrease in egg production, which partially explains the adverse effects of glucocorticoids on production performance in laying hens (El-Lethey et al., 2003; Huang and Shirley Li, 2001). Consistently, the multiple stressors induced by DXM in this study led to a decline in egg production and egg quality. Previous studies have demonstrated that FA supplementation can significantly improve the daily laying rate, egg weight, and egg mass of laying hens (Bagheri et al., 2019; Jing et al., 2014). Correspondingly, FA supplementation alleviated the negative impact of stress on egg production in the current study. These results implied that both the egg laying rate and quality decline, and elevated levels of circulating glucocorticoids may contribute to the decrease in production performance caused by stress.

The liver is the vital metabolic site for chickens, and stressors have been reported to cause liver damage and metabolic disorders; damaging the health and production stability of chickens (Jing et al., 2023). In previous study, chronic heat stress was found to cause hepatic oxidative damage, and induced excessive hepatic lipid deposition in broilers (Jing et al., 2023). Likewise, in the current study, laying hens exposed to DXM, which simulated multiple stressors, developed lipid metabolism disorders and oxidative stress, as evidenced by abnormal increases in serum triglyceride, total cholesterol, and MDA levels. However, dietary FA restored the serum total cholesterol and MDA levels to normal. Consistently, studies have suggested that dietary FA can alleviate alcohol abuse-induced oxidative stress (Ojeda et al., 2016), and reduce fat deposition (Liu et al., 2019). Total bilirubin and total bile acids are associated with liver injury (Huang et al., 2019). In this study, dietary FA downregulated the increased levels of total bilirubin and total bile acids by DXM treatment. This finding is consistent with the oxidative damage to the liver caused by heat stress, as reported by Tang et al. (2022). Our findings support that exposure to stressors increases lipid synthesis, causing liver damage and oxidative stress, which may be one of the internal reasons contributing to the reduction in production performance of laying hens.

To further comprehend the underlying reasons for stress-induced decline in egg production performance, as well as the intrinsic relief mechanism of FA, RNA-seq was employed to detect transcriptional changes in the liver. Among all DEG identified, 8 upregulated and 24 downregulated DEG between the Con and DXM groups were rescued by FA addition. Among these overlapping genes, *CFAP61* mutations primarily affect reproductive functions (Liu et al., 2021), and *CFAP61* exhibits differential expression in obese and non-obese patients (Pei et al., 2017). *LRFN2* has been identified as a gene marker of β-cells (Kang et al., 2023), which has been shown to affect insulin secretion (Taneera et al., 2012). Deficiency in *LRFN2* may also alter calcium influx (Maekawa et al., 2021). *MCM9* functional deficiency is related to ovarian failure and chromosomal instability (Wood-Trageser et al., 2014). *ErbB4* might be a target of estrogen receptors with the potential to reshape the gut microbiota (Ma et al., 2022), and it is one of the necessary genes for melatonin-activated embryo implantation and blastocyst growth (Ivanov et al., 2021). The dynamic changes in the above genes may partially explain the decrease in production performance of the DXM group. Folic acid levels can affect the expression of GCM1 (Li et al., 2021). Deletion of the *LSAMP* gene reduces the sensitivity of mice to stress (Innos et al., 2012). *RXFP1* overexpression has been found to improve oxidative stress and apoptosis in diabetic rats (Sun et al., 2023), and sustain lipid metabolism homeostasis in macrophages (Yang et al., 2024). *UGGT2* protects mouse fibroblasts from endoplasmic reticulum stress induced by saturated lipid via lipid glucosylation (Hung et al., 2022). These aforementioned genes may be involved in the protective effects of FA against oxidative stress, endoplasmic reticulum stress, and lipid metabolism disorders, thereby enhancing production performance. Additionally, pathway enrichment analysis of the DEG between the Con vs. DXM groups revealed enrichment in several pathways, including calcium signaling pathway, transforming growth factor-β signaling pathway, oocyte meiosis, inositol phosphate metabolism, cellular senescence, and mitogen-activated protein kinases (MAPK) signaling pathways. In contrast, the pathways of calcium signaling, steroid hormone biosynthesis, and primary BA biosynthesis were found to be enriched in the DXM vs. FA groups. These results are consistent with the phenotypic findings, which suggest that the reduced egg production rate and quality may be partially attributed to disturbances in oocyte production, calcium metabolism, and oxidative stress.

Metabolic changes are the final result of adaptive and biochemical reactions in the body that occur under multiple stress conditions (Kuang et al., 2021). Serum metabolic signatures of laying hens were analyzed in greater detail. Accumulating evidence suggests that stressors can trigger a swift surge in CORT level in the circulatory system (Alba et al., 2019). The present study revealed a collective phenomenon in which cortexolone levels were elevated in the DXM group, but were downregulated by FA addition (Table S1 and S2), which was consistent with serum CORT phenotype. Among the 38 metabolites up-regulated by the addition of dietary FA, betaine (Chen et al., 2022), indolebutyric acid (Skoczyńska et al., 2024), apigenin (Wu et al., 2021), and hesperidin (Parhiz et al., 2015) have been reported to have antioxidant effects, which may protect from multiple stressors in laying hens. Metabolites between the Con and DXM groups exhibited an enrichment of steroid hormone biosynthesis and calcium signaling pathways, which were associated with reduced production performance (Sun et al., 2024a, Sun et al., 2024b; Wang et al., 2020). In contrast, pathways related to oxidative stress and production performance were also enriched in the DXM vs. FA groups, such as oxidative phosphorylation, steroid hormone biosynthesis, and beta-alanine metabolism. Interestingly, pathways related to butyric acid metabolism and primary BA biosynthesis were enriched in the DXM vs. FA groups. Butyric acid, a functional gut microbial metabolite produced by gut microbiota, functioned as a messenger in the gut–liver axis (Shou and Shaw, 2023). Intestinal microbiota drives butyric acid content and promotes fatty acid oxidation to reduce hepatic steatosis in obesity (Zhang et al., 2023c). Secondary BA are derived from primary BA by specific gut bacteria, and their deficiency induced by gut dysbiosis promotes intestinal inflammation (Sinha et al., 2020). The biological modification of BA by gut bacteria can regulate the homeostasis of gut microbiota and host physiology (Ridlon et al., 2016). Therefore, we want to further explore the interaction between host and gut microbes.

Studies have provided evidence that dietary FA can reduce abdominal fat deposition in broilers via altering gut microbial composition to increase the production of SCFA (Liu et al., 2024). It has been reported that cecum *Fusobacterium*, *Butyricicoccus*, *Faecalibacterium*, and *Megamonas* are positively correlated with dietary FA level, while *Akkermansia*, *Perlucidibaca*, and *Barnesiella* are negatively correlated with FA abundance (Fan et al., 2023). Therefore, this study further investigates whether dietary FA can improve egg production performance of laying hens by affecting gut microbiota. In the heatmap, FA reversed the reduction of *Lactobacillus* in the DXM group. Studies have shown that *Lactobacillus* can enhance the synthesis of butyric acid, which is consistent with the findings from the metabolome-enriched butyric acid pathway. LEfSe analysis revealed that *Mucispirillum* and *Nautella* were the predominant bacteria in the DXM group, while *Clostridium* was the dominant genus in the FA group bacteria at the genus level. *Mucispirillum* is a universal but low-abundance microbiota that has been linked to intestinal inflammation (Herp et al., 2021). Although a few studies have confirmed its pathogenic potential (Zhang et al., 2021), whereas this organism can also promote the health of immunocompetent hosts (Herp et al., 2019). *Nautella* is considered as a beneficial gut bacterium (Lu et al., 2023), and in this experiment, the bacteria exhibited a compensatory increase in response to stresses in the DXM group. *Clostridium butyricum* is a probiotic belonging to the genus *Clostridium* that produces butyric acid, which can regulate the composition of the gut microbiota, inhibit the biotransformation of BA, and promote the synthesis of SCFA (Chen et al., 2020). Moreover, functional prediction analysis revealed that metabolism and fatty acid biosynthesis were enriched in the DXM group, while betaine biosynthesis and retinol metabolism were enhanced in the FA group, suggesting that the stress process was accompanied by lipid metabolism disorder. Dietary FA partially rescued the antioxidant capacity of laying hens through modulation of the intestinal microbial composition and metabolism. These results suggest that there may be interactions between gut microbes and hosts. Therefore, to deconvolute these complex interactions, this study further identified serum BA and cecal SCFA to explore the potential interaction between gut microbiota and the host.

Growing evidence suggests that the gut microbiota and BA play vital roles in maintaining intestinal homeostasis (Yang et al., 2021). Bile acid biotransformation is influenced collaboratively by the host and the gut microbiome, which can also be considered as microbiota-associated metabolites (Thomas et al., 2022). The accumulation of BA can lead to inflammation and liver damage (Mooranian et al., 2019). Bile acids not only regulate the absorption of fat-soluble vitamins, cholesterol, and lipids, but also play a key role in modulating gut epithelial cell proliferation, and microbiome metabolism (Di Ciaula et al., 2017). In this study, the primary BA including CDCA, CA, TCA, and TCDCA, and the secondary BA TDCA were found to be elevated after exposure to stressors, suggesting that inflammation levels and liver damage in laying hens may worsen after stress. However, dietary FA rescued the levels of CA, TDCA, and TCDCA, which may be attributed to the anti-inflammatory and regulatory functions of FA in the gut microbiota (Lu et al., 2022). Taurine, as one of the synthetic substrates of taurine-conjugated BA, was found to be downregulated in the FA group.

In addition to secondary BA, the gut microbiome can also affect host health through its metabolites, such as SCFA (Haarhuis et al., 2022). Short-chain fatty acids play a vital role in multiple biological processes, including intestinal barrier function, host inflammation and lipid metabolism (Zhang et al., 2023a). It has been well-documented that plasma and colonic SCFA levels are associated with metabolic syndromes (Hu et al., 2018); increasing interest in SCFA as signaling molecules between the gut microbiome and the host (Morrison and Preston, 2016). Evidence showed that dietary FA levels can mediate alteration in gut microbiota and SCFA (Mjaaseth et al., 2021). In the current study, the DXM group showed an overall decrease in SCFA levels, while the addition of FA restored the concentrations of acetic acid, propionic acid, and isobutyric acid in the cecum. In patients with chronic kidney disease, there are alterations in the diversity and number of gut microbiomes, which consequently leads to decreased SCFA production (Magliocca et al., 2022). There is evidence to support that SCFA, especially acetic acid and butyric acid, significantly restore the generation of reactive oxygen species and MDA induced by high glucose and lipopolysaccharide (Huang et al., 2017). This suggests that SCFA have a beneficial effect on modulating oxidative stress, which may be implicated in the pathogenesis and progression of multi-stressors. Therefore, the liver–gut axis is closely connected to host health, and regulating this axis holds great potential for managing stress-related disorders.

## 5 Conclusion

**Fig. 4:**
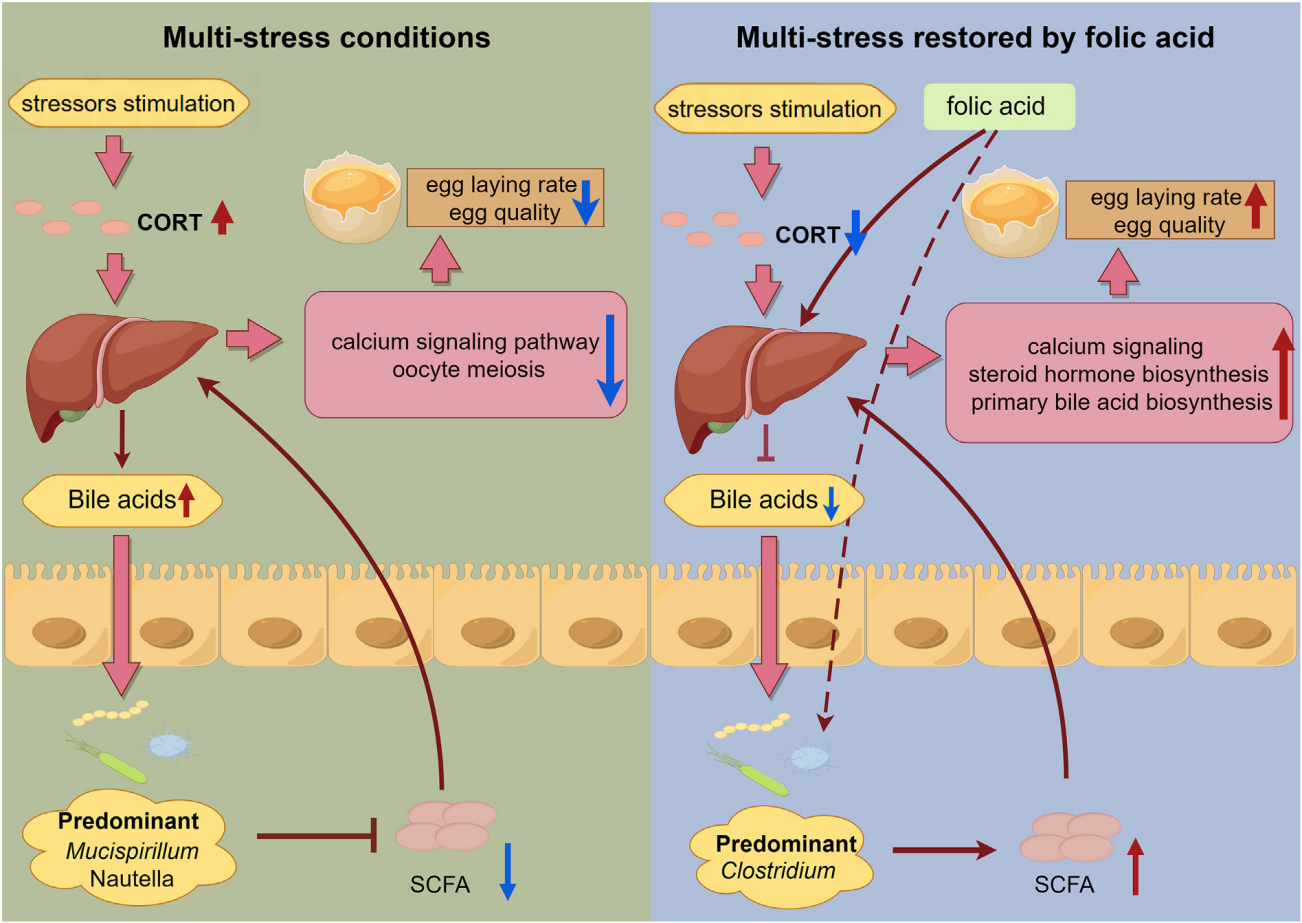
Graphical abstract of the current study, created using Figdraw (www.figdraw.com). This study constructed a multi-stress model for laying hens and systematically elucidated the potential mechanisms of the degradation of production performance caused by stress and the ameliorative effect of folic acid as a promising nutritional additive. Highlighting the prominent role of gut microbe-host interactions in alleviating multi-stressors. CORT = corticosterone; SCFA = short-chain fatty acids.

In summary, this study reveals that the DXM-induced multiple stressors model induces a decline in production performance. This decreased production performance is accompanied by oxidative stress and gut dysbiosis, which promote hepatic lipogenesis and BA production, lower antioxidant capacity, and suppress the production of SCFA. On the contrary, dietary supplementation with FA can increase circulating antioxidant capacity, maintain gut microbiota homeostasis, promote the generation of the SCFA, and suppress the biosynthesis of BA. These results may help to restore gut microorganisms dysbiosis and reduce the occurrence of oxidative stress in laying hens exposed to stressors (Fig. 4), highlighting the prominent role of FA as a promising nutritional intervention through modulating gut–liver axis interactions, alleviating stressor stimuli and maintaining egg production performance in laying hens.

## CRediT authorship contribution statement

**Xi Sun:** Writing – review & editing, Writing – original draft, Software, Conceptualization. **Chaohui Wang:** Data curation. **Sijing Li:** Writing – review & editing. **Xiaoying Liu:** Writing – review & editing, Conceptualization. **Yun Li:** Writing – review & editing. **Yumeng Wang:** Writing – review & editing. **Yuxin Niu:** Writing – review & editing. **Zhouzheng Ren:** Supervision, Project administration. **Xin Yang:** Supervision, Project administration. **Xiaojun Yang:** Supervision, Resources. **Yanli Liu:** Writing – review & editing, Supervision, Project administration, Methodology.

## Data availability statement

The author confirmed that all data underlying the findings in the current study are fully available without restriction from the corresponding author on reasonable request.

## Declaration of competing interest

We declare that we have no financial and personal relationships with other people or organizations that can inappropriately influence our work, and there is no professional or other personal interest of any nature or kind in any product, service and/or company that could be construed as influencing the content of this paper.
