# Supplementary material for: Folic acid alleviates the negative effects of dexamethasone induced stress on production performance in Hyline Brown laying hens
Source: Anim Nutr. 2024 Dec 14;20:54–65. doi: 10.1016/j.aninu.2024.11.011 (PMC11821403; doi:10.1016/j.aninu.2024.11.011)
Supplement: Multimedia component 1 [file mmc1.docx]

**Supplementary Tables**

**Table S1 Differential metabolites between DXM and Con group.**

| **Metabolites** | **VIP^1^** | ***P*-value^2^** |
| --- | --- | --- |
| **Up** |  |  |
| 2-Methoxy-4-vinylphenol | 1.82 | 0.028 |
| Suberic acid | 1.83 | 0.022 |
| Benzoylecgonine | 1.97 | 0.022 |
| Cortexolone | 1.76 | 0.050 |
| 25-Hydroxycholesterol | 1.74 | 0.030 |
| 3-Hydroxyanthranilic acid | 2.32 | 0.002 |
| dTMP | 1.83 | 0.025 |
| (S)-4',5,7-Trihydroxy-6-prenylflavanone | 1.84 | 0.023 |
| Rosmarinic acid | 1.82 | 0.024 |
| Thromboxane B2 | 2.09 | 0.007 |
| Sphingosine 1-phosphate | 2.04 | 0.010 |
| **Down** |  |  |
| Choline | 2.25 | 0.009 |
| Ureidopropionic acid | 2.17 | 0.012 |
| 1,3-Dihydro-(2H)-indol-2-one | 2.34 | 0.006 |
| Chavicol | 1.81 | 0.024 |
| Acetylcholine | 1.91 | 0.018 |
| 4-Hydroxycinnamic acid | 1.88 | 0.019 |
| Acetaminophen | 2.13 | 0.015 |
| Xanthine | 2.10 | 0.012 |
| Nicotine | 1.89 | 0.017 |
| Beta-Tyrosine | 1.83 | 0.023 |
| Indolebutyric acid | 1.72 | 0.040 |
| 12-Hydroxydodecanoic acid | 1.94 | 0.020 |
| Porphobilinogen | 1.72 | 0.035 |
| Ergothioneine | 2.00 | 0.012 |
| Confertifolin | 1.93 | 0.023 |
| 3,3',4'5-Tetrahydroxystilbene | 1.75 | 0.037 |
| Taurohyocholate | 1.80 | 0.026 |
| L-Valine | 1.99 | 0.012 |
| 1-Naphthylamine | 1.83 | 0.023 |
| Salicylic acid | 2.09 | 0.010 |
| Spermidine | 1.89 | 0.034 |
| Hydrocinnamic acid | 2.18 | 0.005 |
| D-Xylose | 2.25 | 0.004 |
| 3,4-Dihydroxyphenylglycol | 1.75 | 0.036 |
| 2-Pyrocatechuic acid | 1.89 | 0.031 |
| Kynurenic acid | 2.05 | 0.009 |
| Citric acid | 1.71 | 0.036 |
| Sebacic acid | 1.95 | 0.029 |
| 5-Hydroxy-L-tryptophan | 2.10 | 0.006 |
| Hexadecanedioate | 1.90 | 0.019 |
| 2-Methoxyestrone | 1.71 | 0.035 |
| Melibiose | 1.78 | 0.029 |
| Luteolin 7-O-beta-D-glucoside | 1.63 | 0.047 |

DXM = dexamethasone.

^1^ Variable importance in the projection (VIP) was obtained from the OPLS-DA model.

^2^ The *P*-value was calculated from Student’s *t* test.

**Table S2 Differential metabolites between FA and DXM group.**

| **Metabolites** | **VIP^1^** | ***P*-value^2^** |
| --- | --- | --- |
| **Up** |  |  |
| D-Fructose | 2.01 | 0.010 |
| Maltol | 1.82 | 0.008 |
| Gamma-glutamylalanine | 1.58 | 0.044 |
| L-Isoleucine | 1.67 | 0.025 |
| N-Formyl-L-methionine | 1.45 | 0.040 |
| 4-Hydroxyproline | 1.70 | 0.025 |
| Uridine | 1.52 | 0.042 |
| Fumaric acid | 1.89 | 0.015 |
| Nicotine | 1.81 | 0.013 |
| Betaine | 1.91 | 0.015 |
| L-Prolinamide | 1.93 | 0.006 |
| 3,3',4'5-Tetrahydroxystilbene | 2.05 | 0.004 |
| L-Lysine | 1.98 | 0.006 |
| Citrulline | 1.62 | 0.027 |
| Indolebutyric acid | 1.98 | 0.006 |
| Carnosol | 1.67 | 0.031 |
| Carbendazim | 2.18 | 0.003 |
| Glyceric acid | 1.57 | 0.048 |
| L-Serine | 1.58 | 0.048 |
| cis-4-Hydroxy-D-proline | 1.65 | 0.026 |
| Protoporphyrinogen IX | 1.67 | 0.021 |
| Homo-L-arginine | 2.01 | 0.039 |
| Gallic acid | 1.65 | 0.022 |
| Hypoxanthine | 1.54 | 0.050 |
| Avermectin A2b monosaccharide | 1.68 | 0.025 |
| Apigenin | 1.72 | 0.016 |
| 2-Hydroxy-6-pentadecylbenzoic acid | 1.65 | 0.045 |
| 3-Oxo-5beta-cholanate | 1.49 | 0.042 |
| Avermectin A1b aglycone | 1.63 | 0.023 |
| cis-9,10-Epoxystearic acid | 1.54 | 0.034 |
| 3-Dehydroecdysone | 1.45 | 0.044 |
| 5alpha-Pregnane-3alpha,20alpha-diol | 1.80 | 0.020 |
| Gentisic acid | 2.77 | 0.000 |
| Hesperidin | 1.47 | 0.045 |
| Telmisartan | 1.69 | 0.021 |
| Melibiose | 1.88 | 0.016 |
| Oleic acid | 1.93 | 0.007 |
| N-Methyltyramine | 2.64 | 0.000 |
| **Down** |  |  |
| Medroxyprogesterone | 1.99 | 0.007 |
| Erythritol | 1.76 | 0.047 |
| Isorhamnetin | 1.69 | 0.024 |
| Ethyl oleate | 1.54 | 0.035 |
| Pantothenol | 1.90 | 0.007 |
| 12,13-DHOME | 2.02 | 0.009 |
| Taurine | 1.55 | 0.046 |
| D-beta-Phenylalanine | 1.93 | 0.012 |
| 11b-PGF2a | 1.61 | 0.036 |
| Alpha-dimorphecolic acid | 1.77 | 0.029 |
| Thromboxane B2 | 1.87 | 0.023 |
| 2-Aminoisobutyric acid | 1.77 | 0.032 |
| Pipecolic acid | 1.66 | 0.018 |
| Cytidine | 1.85 | 0.011 |
| Cortexolone | 1.57 | 0.043 |
| Quinolin-2-ol | 1.88 | 0.007 |
| Cytosine | 1.62 | 0.024 |
| Phosphonoacetate | 1.58 | 0.029 |
| Mesaconate | 1.94 | 0.017 |
| L-Targinine | 1.54 | 0.035 |
| 4-Hydroxy-3-methoxy-cinnamoylglycine | 1.55 | 0.048 |

DXM = dexamethasone; FA = folic acid.

^1^ Variable importance in the projection (VIP) was obtained from the OPLS-DA model.

^2^ The *P*-value was calculated from Student’s *t* test.
